# An Asynchronous Curriculum: Learner Perspectives on Incorporating Asynchronous Learning Into In-Person and Virtual Emergency Residency Didactics

**DOI:** 10.7759/cureus.38188

**Published:** 2023-04-27

**Authors:** Emily L Jameyfield, Semhar Tesfai, Alejandro A Palma, Adriana S Olson

**Affiliations:** 1 Emergency Medicine, University of Chicago, Chicago, USA; 2 Emergency Medicine, John H. Stroger, Jr. Hospital of Cook County, Chicago, USA

**Keywords:** wellness, curriculum development, emergency medicine, zoom, foamed, virtual learning, asynchronous, didactic, residency education

## Abstract

Background

Didactic education in emergency medicine (EM) residencies has been impacted both by the advent of asynchronous learning and by the shift toward virtual, web-based conference education due to coronavirus disease 2019 (COVID-19). Studies have demonstrated the efficacy of asynchronous education, but few have explored resident opinions about how asynchronous and virtual modifications on conference impact their educational experience.

Objective

This study aimed to evaluate resident perceptions of both asynchronous and virtual modifications to a historically in-person didactic curriculum.

Methods

This was a cross-sectional study of residents of a three-year EM program at a large academic center where a 20% asynchronous curriculum was implemented in January 2020. A questionnaire was administered online with questions assessing how residents perceived their didactic curriculum with regard to convenience, retention of information, work/life balance, enjoyability, and overall preference. Questions compared resident opinions of in-person vs. virtual learning, as well as how the substitution of one hour of asynchronous learning impacted residents' perception of their didactics. Responses were reported on a five-point Likert-type scale.

Results

A total of 32 out of 48 residents (67%) completed the questionnaire. When virtual conference was compared to in-person conference, residents favored virtual conference with regard to convenience (78.1%), work-life balance (78.1%), and overall preference (68.8%). They favored in-person conference (40.6%) or felt that the modalities were equivalent (40.6%) with regard to retention of information and favored in-person conference with regard to enjoyability (53.1%). Residents felt that the addition of asynchronous learning to their curriculum increased subjective convenience, work-life balance, enjoyability, retention of information, and overall preference, regardless of whether synchronous conference was virtual or in-person. All 32 responding residents were interested in seeing the asynchronous curriculum continue.

Conclusion

EM residents value the addition of asynchronous learning to both in-person and virtual didactic curricula. Additionally, virtual conference was favored over in-person conference with regard to work/life balance, convenience, and overall preference. As social distancing restrictions continue to ease post-COVID-19 pandemic, EM residencies may consider adding or maintaining asynchronous or virtual components to their synchronous conference schedule as a means to support resident wellness.

## Introduction

As mandated by the Accreditation Council for Graduate Medical Education (ACGME), every emergency medicine (EM) residency training program includes five hours per week of protected core education time. Most EM residencies block this time as one half-day of mandatory conference time each week [1]. 

Over the past decade, two events have significantly impacted the structure of weekly didactic education for EM residents: the advent of free open access medical education (FOAMed) resources and the SARS-CoV-2 (coronavirus disease 2019 (COVID-19)) global pandemic. With FOAMed, there is a comprehensive, ever-growing bank of high-quality educational resources available online for review of EM topics. This has promoted increased popularity of individualized interactive instruction (III), also known as asynchronous learning (AL), as a partial substitute for standard didactics. Per the ACGME, asynchronous instruction can comprise up to 20 percent of an EM program’s mandatory didactic time [1]. The exact content of a program’s asynchronous component is adaptable, as long the program meets specific parameters set forth by the ACGME to assure curriculum effectiveness.

Due to the COVID-19 global pandemic, beginning in March 2020, social-distancing safety measures required an unprecedented nationwide shift from in-person, proximate didactics toward virtual, distance education. Prior studies have explored how these two shifts in EM education, the shift from synchronous learning to AL [2,3] and the shift from in-person to virtual didactics [4], impact resident learning. Less studied is the modern overlap of the two modalities: how AL is perceived by residents in a virtual curriculum compared to an in-person curriculum. It has been shown that AL reliably prepares residents for academic success in that an asynchronous format is non-inferior to lecture-based didactics in preparing residents for their in-training exam [2]. Adoption of the AL learning format is so widespread that the Council of Emergency Medicine Residency Directors (CORD) released a Guide to Best Practices in 2019 [1]. However, previous studies have not focused on how residents feel about these curricular modifications, calling into question whether the effort dedicated toward developing AL curricula is worthwhile for programs and learners.

This study aimed to directly evaluate resident perceptions of an asynchronous curriculum after it began in January 2020. We hypothesized that a combination of in-person didactic conference and AL would be preferred by learners as compared to in-person didactic conference alone. Shortly after initiating the asynchronous curriculum, the COVID-19 pandemic forced a transition to virtual conference. All in-person didactics began taking place over Zoom as a virtual learning space. We recognized some inherent parallels between virtual and asynchronous learning, such as the ability to learn from home, and thereby chose to also question residents about their perception of virtual vs. in-person learning. We hypothesized that residents would report subjective benefits to virtual learning compared to in-person learning and that they would still positively perceive the existence of AL within the virtual learning environment.

This article was previously presented as a poster at the 2021 CORD Academic Assembly on April 12, 2021.

## Materials and methods

Overview

This was a cross-sectional study with two objectives: First: to explore how residents perceive virtual didactics compared to in-person didactics and second: to explore how residents perceive the inclusion of AL in their didactic curriculum. A questionnaire was administered online to residents of a three-year academic EM program where a new asynchronous curriculum was established in January 2020. The questionnaire, administered in October 2020, assessed how residents from the classes of 2020-2022 perceived in-person and virtual didactics, both with and without AL. Ethical approval via IRB exemption was granted by the Institutional Review Board of the University of Chicago, IRB exemption number IRB20-1676.

Study site curriculum

At the studied institution, conference was historically composed of five hours of in-person lectures once per week. In January 2020, we piloted a new asynchronous curriculum. Didactic modules were already organized based on the organ system. For each 3-12 week module, residents were provided a curated list of high-quality FOAMed resources published within the last five years. Each resource was associated with an anticipated length of time required for review. Each week, residents were responsible for reading, watching, or listening to enough resources to comprise one full hour’s worth of time. Compliance was tracked via a spreadsheet where residents logged their resources of choice and wrote three learning points they had taken away from their resources. Learning retention was monitored and reinforced by in-conference end-of-module quiz games.

Questionnaire design

The questionnaire asked residents to consider five subjective elements of their didactic experience: convenience, retention of information, work/life balance, enjoyability, and preferability (Table 1). These variables were chosen via group consensus by the four authors (two residents, two faculty members) with feedback from a group of EM faculty medical educators at their institution as a holistic representation of what brings subjective “value” to an EM resident’s educational experience. 

**Table 1:** Resident perceptions of the asynchronous learning questionnaire

Question	Stem	Responses				
1a	How convenient is five hours of in-person conference compared to five hours of remote/zoom conference per week?	Very convenient	Somewhat convenient	Equivalent	Somewhat inconvenient	Very inconvenient
1b	How convenient is one hour of asynchronous learning + four hours of in-person conference compared to five hours of in-person conference per week?	Very convenient	Somewhat convenient	Equivalent	Somewhat inconvenient	Very inconvenient
1c	How convenient is one hour of asynchronous learning + four hours of remote/zoom conference compared to five hours of remote/zoom conference per week?	Very convenient	Somewhat convenient	Equivalent	Somewhat inconvenient	Very inconvenient
2a	How advantageous for your retention of information is five hours of in-person conference compared to five hours of remote/zoom conference per week?	Very advantageous	Somewhat advantageous	Equivalent	Somewhat disadvantageous	Very disadvantageous
2b	How advantageous for your retention of information is one hour of asynchronous learning + four hours of in-person conference compared to five hours of in-person conference per week?	Very advantageous	Somewhat advantageous	Equivalent	Somewhat disadvantageous	Very disadvantageous
2c	How advantageous for your retention of information is one hour of asynchronous learning + four hours of remote/zoom conference compared to five hours of remote/zoom conference per week?	Very advantageous	Somewhat advantageous	Equivalent	Somewhat disadvantageous	Very disadvantageous
3a	How is your work/life balance with five hours of in-person conference compared to five hours of remote/zoom conference per week?	Much better	Somewhat better	Equivalent	Somewhat worse	Much worse
3b	How is your work/life balance with one hour of asynchronous learning + four hours of in-person conference compared to five hours of in-person conference per week?	Much better	Somewhat better	Equivalent	Somewhat worse	Much worse
3c	How is your work/life balance with one hour of asynchronous learning + four hours of remote/zoom conference compared to five hours of remote/zoom conference per week?	Much better	Somewhat better	Equivalent	Somewhat worse	Much worse
4a	How enjoyable is five hours of in-person conference compared to five hours of remote/zoom conference per week?	Much more enjoyable	Somewhat more enjoyable	Equivalent	Somewhat less enjoyable	Much less enjoyable
4b	How enjoyable is one hour of asynchronous learning + four hours of in-person conference compared to five hours of in-person conference?	Much more enjoyable	Somewhat more enjoyable	Equivalent	Somewhat less enjoyable	Much less enjoyable
4c	How enjoyable is one hour of asynchronous learning + four hours of remote/zoom conference compared to five hours of remote/zoom conference?	Much more enjoyable	Somewhat more enjoyable	Equivalent	Somewhat less enjoyable	Much less enjoyable
5a	How preferable is five hours of in-person conference compared to five hours of remote/zoom conference per week?	Much more preferable	Somewhat preferable	Equivalent	Somewhat less preferable	Much less preferable
5b	How preferable is one hour of asynchronous learning + four hours of in-person conference compared to five hours of in-person conference per week?	Much more preferable	Somewhat preferable	Equivalent	Somewhat less preferable	Much less preferable
5c	How preferable is one hour of asynchronous learning + four hours of remote/zoom conference compared to five hours of remote/zoom conference per week?	Much more preferable	Somewhat preferable	Equivalent	Somewhat less preferable	Much less preferable
6	How interested are you in having the asynchronous curriculum continue?	Very interested	Somewhat interested	Neutral	Somewhat disinterested	Very disinterested
7	What class year are you?	Class of 2023 (pilot group)	Class of 2022	Class of 2021	Class of 2020	
8	Any additional thoughts, suggestions, or concerns about the UCEM asynchronous curriculum:					

The 18-item questionnaire began with five sets of three items a piece, where each set corresponded to one of the five aforementioned elements. Items all used a five-point Likert-type scale with varying descriptive anchors. The first item (item A) in each set asked residents to compare in-person didactics with virtual didactics. The second and third items (items B and C) in each set paralleled one another, asking residents to compare one hour of asynchronous didactics + four hours of in-person (B) or virtual (C) synchronous didactics to five hours of equivalent synchronous didactics.

Of note, at the studied institution, the AL curriculum started prior to the pandemic, and thereby prior to any virtual conference. For this reason, surveyed residents had never partaken in five full hours of virtual didactic conference. However, as it was unclear at the time both how successful the AL curriculum would be as well as how indefinitely social-distancing restrictions would last, the questionnaire asked residents to extrapolate on their known experiences and consider the differences between five hours of in-person and five hours of virtual conference time, as shifting to a five-hour virtual model was a realistic possibility.

Validity

The questionnaire (Table 1) was assembled via an iterative review process, in accordance with the survey checklist and questionnaire guidelines outlined in Academic Medicine [5,6]. To ensure that the content of each item was relevant to the assessment, five medical education experts familiar with the institution’s curriculum reviewed the questionnaire prior to its administration and provided feedback. Subsequent to their feedback, questionnaire wording was revised to allow for maximum clarity. To protect responses from bias, the questionnaire was made anonymous, and the study team was blinded to the identity of participating residents. Attention was paid to the response process by piloting the questionnaire with residents in the class of 2023 (56% participation, 9/16 residents) at the studied institution. These residents had not participated in the original curriculum and thus were ineligible for full participation in the study, but their feedback was used to test the questionnaire’s validity. Participants were given the opportunity to provide anonymous suggestions regarding question clarity. No comments were provided, and thus the same version of the questionnaire was provided to the classes of 2020-2022.

Data collection

The questionnaire was finalized as a Google form. It was distributed via email listserv to all residents from the classes of 2020, 2021, and 2022 in mid-October 2020. Residents were given three weeks to fill out the form, with two emailed reminders to complete the questionnaire during that time. There was no incentive offered for completion of the form. Out of thirty-three total submissions, two submissions had an identical time stamp and responses including an identical narrative comment. This was interpreted as a technical error and one of the duplicative responses was not included in the final data set.

Data interpretation

The explored hypotheses rested on understanding whether residents favored one learning modality over another as they pertained to five different aspects of subjective value. The collected data were interpreted in two segments so as to address the two study objectives. 

Item A from every set in the questionnaire pertained to the first objective: how residents perceive virtual didactics compared to in-person didactics. For clarity of interpretation and discussion, the Likert-type item responses were lumped together on either side of the middle anchor which was “equivalent.” Responses of in-person didactics being “very” or “somewhat” more convenient or advantageous for retention of learning and “much” or “somewhat” better for work/life balance, more enjoyable, or more preferable were combined to express how many total residents favored in-person learning. Responses of in-person didactics being “very” or “somewhat” inconvenient or disadvantageous for retention of learning and “much” or “somewhat” worse for work/life balance, less enjoyable, or less preferable were combined to express how many total residents favored virtual learning. Frequencies of responses were reported in percentages.

Items B and C from every set in the questionnaire pertained to the second study objective: examining how residents perceive the inclusion of AL in their didactic curriculum. For clarity of interpretation, the narrative anchor responses for items B and C from each question were converted to a numeric scale, where higher values indicated increased subjective favorability of four hours + one hour of AL compared to five hours of synchronous learning, and a value of 3 was “equivalent.” As a reflection of the spectrum of favorability, the responses for items B & C across the five question sets were interpreted as interval data. Means were reported as the measure of central tendency. Frequencies for each item response were reported in percentages without lumping responses.

A two-tailed paired t-test was performed to examine whether the independent variable of in-person vs. virtual learning significantly impacted how residents perceived the addition of AL to their curriculum.

## Results

A total of 32 out of 48 residents (67%) completed the questionnaire. Based on the nature of the class year, the amount of total pre-questionnaire exposure to each didactic structure varied. All participating classes experienced a minimum of six months of in-person didactics without AL, 2.5 months of in-person didactics with AL, and a minimum of 3.5 months of virtual didactics with AL (Table 2).

**Table 2:** Class year and didactic experience of residents completing the questionnaire AL: Asynchronous learning

	Total respondents (%) N = 32	Months with fully in-person didactics prior to study	Months with in-person + AL prior to study	Months with virtual + AL prior to study
Class of 2020	8 (25)	30	2.5	3.5
Class of 2021	14 (44)	18	2.5	6.5
Class of 2022	10 (31)	6	2.5	6.5

**Figure 1:**
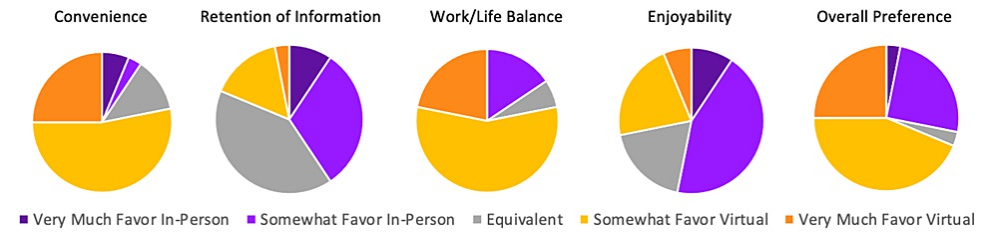
Resident perspective regarding didactics as five hours/week of in-person vs. virtual education

First, we examined resident perspectives on in-person conference compared to virtual conference independent of the addition of AL (Figure 1). With regard to convenience, 78.1% of residents favored virtual conference, 9.4% favored in-person conference, and 12.5% found them to be equivalent. With regard to retention of information, 18.8% of residents favored virtual conference, 40.6% favored in-person conference, and 40.6% found them to be equivalent. With regard to work/life balance, 78.1% of residents favored virtual conference, 15.6% favored in-person conference, and 6.3% found them to be equivalent. With regard to enjoyability, 28.1% of residents favored virtual conference, 53.1% favored in-person conference, and 18.8% found them to be equivalent. With regard to overall preference, 68.8% of residents favored virtual conference, 28.1% favored in-person conference, and 3.1% found them to be equivalent.

**Figure 2:**
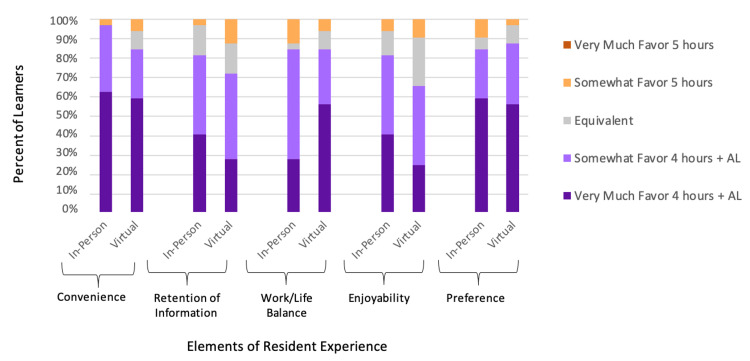
Resident learning experience with regard to five hours of synchronous didactics vs. four hours of synchronous didactics + one hour of asynchronous learning, during both in-person and virtual curricula AL: Asynchronous learning

Next, we examined resident perception of the addition of AL to their curriculum. Across all five questioned elements of subjective “value”, residents favored four hours of in-person or virtual conference + one hour of AL more than five hours of in-person or virtual conference, with Likert-type scale means ranging from 3.81 to 4.56 (Table 3). There were no residents who strongly favored five hours of in-person or virtual didactics without AL with regard to convenience, retention of information, work/life balance, enjoyability, or preference (Figure 2). 

**Table 3:** Perceived resident favorability of five hours synchronous vs. four hours synchronous + one hour asynchronous learning in the setting of in-person vs. virtual conference *: significant (p < .05), AL: asynchronous learning; SD: standard deviation

		Paired difference (N = 32; df = 31)			
5 hours vs. 4 hours + 1 hour AL		Mean (SD)	Mean	t	Sig (2-tailed)
Convenience	In-Person vs. IP + AL	4.56 (.67)	.19	1.0	.325
	Virtual vs. Virtual + AL	4.38 (.91)			
Retention of information	In-Person vs. IP + AL	4.19 (.82)	.31	1.72	.096
	Virtual vs. Virtual + AL	3.88 (.98)			
Work/life balance	In-Person vs. IP + AL	4.00 (.92)	-.34	-1.41	.169
	Virtual vs. Virtual + AL	4.34 (.90)			
Enjoyability	In-Person vs. IP + AL	4.16 (.88)	.34	1.93	.062
	Virtual vs. Virtual + AL	3.81 (.93)			
Preference	In-Person vs. IP + AL	4.34 (.97)	-.06	-.28	.782
	Virtual vs. Virtual + AL	4.41 (.80)			

When testing whether favorability of the addition of AL varied in the context of in-person conference compared to virtual conference, no significant differences (p < .05) were found for any of the five surveyed elements (Table 3). 

All 32 responding residents reported that they were either somewhat interested (12%) or very interested (88%) in seeing the asynchronous curriculum continue.

## Discussion

As hypothesized, residents at the studied program clearly value the addition of AL to their didactic curricula. Residents favored the substitution of one hour of AL for synchronous learning across all five explored elements of their educational experience including convenience, retention of information, work/life balance, enjoyability, and preference. These findings persisted regardless of whether the conferences were in-person or virtual. This study supports what many intuitively believe to be true, which is that residents value having agency over their own learning and appreciate flexibility within their didactic curricula. Previous studies and articles have outlined for EM programs how best to incorporate AL into conference time [1,2,3]. Skeptics of AL question whether it is educators’ responsibility to support resident wellness over their education. However, it has already been shown that program adoption of AL into didactic curricula results in non-inferior ITE performance [3]. This study supports programmatic efforts dedicated to incorporating AL into synchronous didactic curricula, as doing so may have meaningful impacts on the learner experience and thereby promote a healthy balance of education and wellness.

In terms of virtual vs. in-person conference, the authors hypothesized that residents would favor virtual conference across the five examined elements of subjective value. While residents did favor virtual conference in relation to convenience, work/life balance, and overall preference, they favored in-person didactics more in relation to retention of information and enjoyability. Further research should be dedicated toward exploring how well learners objectively retain information following virtual compared to in-person conference since learners in this study subjectively felt their retention was better in-person than virtually.

A recent study demonstrated that during virtual conference residents participated in nearly twice as many non-conference-related activities per hour, such as exercising or washing dishes than they did during in-person conference [4]. This ability to multi-task likely contributes to the subjective edge that virtual conference has over in-person conference in relation to work/life balance and convenience. As many forms of AL such as videos or podcasts similarly allow for easier multitasking for learners, a future study looking at how many residents multitask while completing their AL education may offer additional insight into how and why AL is perceived so positively by learners. 

The degree to which the flexibility provided by virtual and asynchronous learning supports resident wellness should be acknowledged by programs as social distancing restrictions continue to ease. Programs that did not initially incorporate AL into their didactic curricula may consider the addition of AL and/or the inclusion of occasional virtual conference days as a way to support resident preference and wellness moving forward. 

Limitations

The studied AL curriculum was based on best practices as well as previously implemented curricula at other sites [1,3]. However, the study was still single-site, and thus, the resident opinions all reflect one specific way that their residency program implemented both the asynchronous and virtual didactic curricula. This is significant, because though the ACGME sets guidelines, the specific FOAMed resources chosen are institution-specific [1]. A future study across multiple institutions would give more robust insight into resident opinion. The study was also limited in its scope. The questionnaire evaluated learner perception of AL and in-person vs. virtual curricula but did not discuss or evaluate higher order outcomes such as educational efficacy. Future studies could look at how the intersection of AL and virtual learning impacts in-service scores, practice oral board scores, or other objective measures of knowledge and preparedness [2].

One limitation of the questionnaire is that residents were at no point asked to directly compare four hours of in-person learning + one hour of AL with four hours of virtual learning + one hour of AL. The intent behind asking residents to compare five hours of in-person conference with five hours of virtual conference was to have residents directly compare the two modalities irrespective of the addition of AL, in case program didactics should ever convert to a fully virtual format without AL. However, since residents had never participated in five full hours of virtual learning, answering the questionnaire inherently necessitated a degree of extrapolation by the residents.

Additionally, by nature of the structure of the institution’s asynchronous curriculum, each resident chose their own AL educational materials from a provided list of options each week. It was impossible to control for all potential differences in the chosen resources, which may have contributed to varying resident experiences.

One other limitation that should be acknowledged is the timing of the questionnaire. The asynchronous curriculum and virtual curriculum changes discussed happened at the height of the COVID-19 pandemic, and the questionnaire was distributed and completed in November 2020, which was still pre-vaccine availability. It is possible that resident trepidation surrounding return to in-person conference may have contributed to their responses. Certain elements of virtual vs. in-person and synchronous vs. asynchronous learning persist regardless of cultural atmosphere, but it may be beneficial to repeat the study in today’s evolving post-pandemic climate.

## Conclusions

EM residents at the studied institution favored the substitution of 20% AL for standard didactic education regardless of whether conference was virtual or in-person. Additionally, virtual conference was favored over in-person conference with regard to work/life balance, convenience, and overall preference. As social distancing restrictions continue to ease post-COVID-19 pandemic, EM residencies may consider adding or maintaining asynchronous or virtual components to their synchronous conference schedule as a means to support resident wellness.
